# What contributes to older Korean American immigrants becoming frail? A qualitative study

**DOI:** 10.1186/s12877-024-05565-2

**Published:** 2024-12-04

**Authors:** Hae Sagong, Myoung-Gi Chon, Pao-Feng Tsai, Ah Ram Jang

**Affiliations:** 1 College of Nursing, Auburn University, 710 South Donahue Drive, Auburn, AL USA; 2 School of Communication and Journalism, Auburn University, Auburn, AL USA; 3 Department of Nursing, Yeoju Institute of Technology, Yeoju, Republic of Korea

**Keywords:** Korean american, Immigrants, Health literacy, Limited english proficiency, Frailty

## Abstract

**Background:**

Frailty is prevalent in older adults and represents a dynamic condition that can improve with early detection and interventions focused on influencing factors. Older immigrant populations, marked by diverse cultural backgrounds, distinct health beliefs and behaviors, language barriers, and low health literacy, require a focused exploration of factors influencing frailty. This study examines the perceived factors affecting frailty among older Korean American immigrants (OKAIs).

**Methods:**

A focus group interview was conducted with nine OKAIs (five frail/prefrail and four robust) in Alabama, supplemented by demographic and health-related surveys. Semi-structured questions addressed health literacy, physical activity, nutrition, and mental health. Data analysis included both descriptive and content analysis methods.

**Results:**

Participants displayed notable differences in health literacy scores between Korean and English assessments. Content analysis highlighted that social activities, health behaviors, and healthcare utilization for OKAIs were shaped by distinct environmental and healthcare contexts compared to those in Korea. Limited English proficiency and access to healthcare resources further influenced their experiences. Participants expressed a preference for Korean healthcare providers, interpreters, and traditional food options to maintain well-being.

**Conclusion:**

Interventions aimed at preventing and managing frailty among OKAIs should consider their unique characteristics and challenges to improve effectiveness.

## Background

Frailty is a state in which a person is vulnerable to stressors and has decreased physiological resilience, resulting in increased disability, adverse health outcomes, morbidity, and mortality. It is often assessed and diagnosed based on an individual’s physical function or inactivity and weight loss [1, 2].

Frailty is prevalent in community-dwelling older adults, with estimates ranging from 4 to 24% depending on the study or measurement used [3–5], and is more prevalent among women and racial/ethnic minorities [6, 7]. Factors contributing to frailty include physical, nutritional, cognitive/psychological, and social factors as well as aging and multimorbidity [8, 9]. In addition, socioeconomic statuses such as education, income, and level of health literacy were also found to be determinants of frailty [10, 11]. Unlike aging, frailty is a dynamic state that can improve over time with early detection and proper intervention [12, 13]. Therefore, it is essential to identify the influencing factors of frailty based on the specific population to develop a structured frailty intervention that effectively reduces and prevents frailty.

European studies have shown that racial/ethnic minorities, including individuals with migrant backgrounds, are frailer than native populations [14–16]. They are considered vulnerable groups who need special attention. The factors contributing to immigrant populations’ higher prevalence of frailty are not well understood; however, studies have revealed that groups from low- or middle-income countries are more likely to be frail [16, 17]. This finding is not solely due to socioeconomic inequalities. Language barriers and low levels of acculturation among immigrants may also contribute [18], leading to restricted access to healthcare and structural obstacles, including discrimination [14, 15]. Consequently, these results can lead to psychological distress [19], low self-rated health [18], low health literacy [20], and health disparities [21], all of which accelerate the development of frailty over the long term [22].

In the United States, initiatives have been introduced to recognize frailty within racial and minority populations [6, 23], as previous studies on frailty have primarily focused on sex and age groups [3]. Nevertheless, the majority of research conducted in the US has primarily focused on White and African-American communities, often aggregating various other racial and ethnic groups. This approach makes it challenging to accurately estimate frailty prevalence in specific racial and ethnic groups other than white and African American and to identify influencing factors [6, 7, 23]. For example, despite Asians being known as highly heterogeneous, with varying health outcomes [24], efforts aimed at investigating frailty and factors that led to frailty among Asian ethnic subgroups with a migration background are notably limited. In particular, older immigrants face distinct challenges compared to non-immigrant older adults due to their tendency to maintain low levels of acculturation and preserve their ethnic characteristics [25]. These factors render them more vulnerable to frailty. Therefore, it is imperative to identify the race and ethnicity-specific factors that contribute to frailty among older immigrants, recognizing the distinct challenges they face.

In light of the prevailing circumstances, this study seeks to investigate the determinants of frailty among older Korean American immigrants (OKAIs) residing in suburban regions with a significant number of Korean American immigrants compared to other suburban regions but a scarcity of Korean healthcare facilities, including Korean clinics or healthcare providers, unlike many metropolitan cities. Through a focus group interview with OKAIs, considering their ethnicity and cultural background, this study will provide insights into the specific factors influencing frailty in this population. The data from this study can inform the development of targeted interventions to prevent and manage frailty.

## Methods

### Study design and sample

This is a qualitative study [26] aimed at investigating the factors that OKAIs perceive as influencing frailty. The study received ethical approval from the Institutional Review Board (No. 22–262 EX 2206). A focus group interview was conducted to collect OKAIs’ points of view, since little was documented about them [27]. A sociodemographic and health-related characteristic survey was also conducted before the interview to understand participants’ characteristics including demographics, health status, and others.

A convenience sample of the OKAIs was recruited from Korean churches in Lee County, Alabama, and an online Korean community for Lee County residents. Lee County is home to one of the largest investments made by Korean automobile companies, leading to one of the highest Korean populations in Alabama [28]. The inclusion criteria for participants were (1) 65 years and older; (2) born in South Korea and moved to the U.S. more than a year ago; (3) able to speak and understand Korean. Focus group interview size is recommended to be from 6 to 12 participants per group [29] and this study recruited 10 eligible participants who agreed to participate. Of these, 9 completed the interview as one focus group.

### Measures and data collection

Data collection started after participants consented to participate. Informed consent was obtained from all individual participants included in the study. Before starting the focus group interview, a paper survey was implemented to gather general information about participants. The survey includes demographic characteristics (age, gender, education attainment, marital status, period of residency in the U.S., level of English skill), health literacy, and health-related information (frailty, physical activity, nutrition, cognitive function, depression). Health literacy was measured with the Korean version of the Health Literacy Survey-12 Questionnaires (HLS-EU-Q12) [30] and REALM-SF [31] in English and Korean versions. Frailty was measured with FRAIL [32], physical activity was measured with Global Physical Activity Questionnaire (GPAQ) [33], and nutrition was measured with Mini Nutritional Assessment (MNA) [34]. Cognitive function and depression were measured by using the Korean-Mini-Mental State Examination (K-MMSE) [35] and the Geriatric Depression Scale-Short form (SGDS) [36]. An investigator-designed questionnaire was used to collect information regarding perceived health status, regular health check-ups, smoking, drinking, race of primary doctor, considerations when choosing primary care, and influencing factors of frailty. Participants were given 40 min to answer survey questionnaires, with instruction by the principal investigator (PI) in Korean.

For the focus group interview, semi-structured questions were asked about four factors that are related to frailty: health literacy, physical activity, diet, and mental health to provide opportunities to derive more elaborate responses [37]. The interview was conducted in a quiet room in one of the Korean churches in Lee County, Alabama. The moderator used semi-structured questions to lead the interview (Table 1) and encouraged participants to share their thoughts and feelings about the facilitators and barriers to each of the four factors related to frailty. The interview lasted for approximately three hours, at which point it reached full saturation of data. This was evidenced by the repetition of themes and the point at which no new information or insights emerged from the discussion. The interviews were recorded with each participant’s permission, and the research assistant took notes throughout, including core messages from each participant and summaries, using Microsoft Word. The research assistant also monitored the recording status and provided a debrief with a moderator. Afterward, the recorded interviews were fully transcribed by the research assistant and reviewed and finalized by two researchers, including the moderator and principal investigator, along with one research assistant.

**Table 1:** Interview questionnaires

Stage of interview		Questions
Opening		Before getting started, we will take a moment to introduce ourselves.
Introductory		What makes you become frail while living in the U.S.?
Key questions	Health literacy	What makes (healthcare system, language, etc.) you understand health-related information more?
		What is the main barrier to understanding health-related information?
	Physical activity	What makes you do physical activity more?
		What’s the main problem that bans your physical activity?
	Nutrition	What makes you have a healthy diet?
		What bans you from doing a healthy diet?
	Mental health	What makes you get mental health care?
		What’s the barrier to mental health care?
Closing		Please tell us if there are any questions or thoughts you like to share.

### Data analysis

Sociodemographic and health-related survey data were analyzed through descriptive statistics, including means, standard deviations, and numerical counts, to illustrate the participants’ characteristics. Qualitative content analysis was conducted for the interview data. Content analysis is a widely used qualitative research analysis technique that provides knowledge and understanding of the phenomenon under study by organizing, interpreting, and categorizing themes from the data [38, 39]. This study used an inductive approach, which generalizes conclusions from interview data by extracting characteristics and repeated concepts through repeated review [40]. Once a research assistant transcribed the full interview with recordings, the PI reviewed its accuracy. Two trained coders with extensive knowledge of the health of older Korean adults reviewed the transcription thoroughly and repeatedly until they were able to fully understand and document meaningful data from the interview. Then, sentences or keywords that were relevant to the questions were elicited and labeled as codes. During the coding process, to avoid any potential biases, the third person who is not living in the US in the research team reviewed the code. Back-and-forth reviews and discussions were made throughout the process to ensure rigor. The codes were organized into sub-categories, which were gathered into categories and finalized by all research team members along with one external qualitative research expert [39]. When translating the finalized codes and verbiage into English, two bilingual researchers reviewed and agreed on the translation. The final translated findings were then reviewed by an English speaker, who provided feedback on any misunderstandings.

## Results

### Characteristics of participants

Table 2 provides descriptive statistics of sociodemographic and health-related survey results of the nine study participants, comprising eight females and one male. Participants’ mean age was 69 (SD = 4.77) years old. Four had an educational attainment of at or below high school education and five completed college or above. The mean period of residency in the U.S. was 19.33 (SD = 13.60) years with a wide range of 4 to 48 years. Most of the participants were married (*n* = 7) and had limited English proficiency (*n* = 7). Regarding frailty, four participants considered their health robust (*n* = 4) and five described themselves as pre-frail or frail (*n* = 5). Using the same health literacy measurement tool (REALM-SF) but in different languages, the English version’s mean score was 1.89 ± 2.21, while the Korean version’s mean score was 6.78 ± 0.44, showing significant differences, with the Korean version being significantly higher. The other health literacy measurement, the Korean version of HLS-EQ-12, showed a mean score of 2.39 ± 0.49, indicating a moderate level of health literacy. The mean physical activity score (GPAQ) was 600.00 ± 714.98 METs (metabolic equivalent)/week. Five participants were at-risk of malnutrition and five participants showed fair or bad perceived health status. The mean cognitive function score (K-MMSE) was 28.78 (SD = 1.64) and the score of depressive symptoms (GDS) was 3.33 (SD = 2.87), which shows no cognitive impairment or depressive symptoms, respectively. Most participants have regular health checkups (*n* = 7), and none of the participants smoke or drink. When asked about the doctor they visited, four participants only visited a Korean doctor, and five participants said that the availability of a Korean doctor was the most important consideration in choosing a primary doctor. The most frequently identified influencing factors of frailty were limited English proficiency, followed by health literacy, diet, hospital accessibility, and mental health.

**Table 2:** Characteristics of participants (*N* = 9)

Variable	Categories	Min-max	M ± SD or *n*
Age		65–77	69 ± 4.77
Education	Elementary		1
	Middle school		2
	High school		1
	Above college		5
Period of residency in the U.S. (years)		4–48	19.33 ± 13.60
Marriage status	Married		7
	Divorced/endowed		2
English proficiency	Limited		7
	Fluent		2
Frailty	Robust		4
	Pre-frail		4
	Frail		1
Health literacy	REALM-SF English (0–7)	0–6	1.89 ± 2.21
	REALM-SF Korean (0–7)	6–7	6.78 ± 0.44
	HLS-EQ-12 (1–4)	1.67–3.33	2.39 ± 0.49
Physical activity (GPAQ, METs/week)		0–2160	600.00 ± 714.98
Nutrition	At-risk of malnutrition		5
	Well-nourished		4
Cognitive function (K-MMSE)		25–30	28.78 ± 1.64
Depressive symptoms (SGDS)		1–15	3.33 ± 2.87
Perceived health status	Good		4
	Fair		3
	Bad		2
Regular health check-up	Yes		7
	No		2
Smoking or Drinking	No		9
Race of primary doctor	Korean		4
	Others or have no primary doctor		5
Considerations for choosing a primary doctor	Availability of Korean		5
	Distance		3
	Others		1
Influencing factors of frailty	Limited English proficiency		4
	Limited health literacy		3
	Diet		2
	Hospital accessibility		2
	Mental health		2

### Qualitative analyses

Seven categories and 22 sub-categories were derived (Table 3) from 80 codes. All quotes are marked with a “P” followed by participant numbers and basic demographics (sex, age, and marital status) at the end of each quote to provide clear distinction.

**Table 3:** Factors influencing older Korean American immigrants’ frailty

Codes	Sub-category	Category
Needs a lot of paper works before the examination Takes a long time to schedule an appointment Have no health insurance Reduced benefit of Medicare	Low accessibility to healthcare institutions	Limited available health resources
No Korean doctor Lack of health seminars for Korean	Lack of health resources for Koreans	
Limited exercise center/equipment for seniors	Lack of resources for older adults	
Unwanted health information on Youtube Getting health information from close friends Unreliable health information from the internet Follow unproven remedies Feeling no necessity to find health information Mainly finding health information via the internet Can’t rely only on doctor’s opinion	Indiscriminating health information	
Reluctant to visit a clinic Accepting losses Frustrated/stressed/Misunderstanding in communication with health staff Have mistrust in American doctor Need cultural understanding between staff and immigrants	Expecting the loss/discrimination	Limited English skill
Ask for interpretation from children or friends Needs an official health interpreter Understand English by using a translator Searching health information in Korean to understand	Need of an interpreter	
Consumes protein intentionally Early dinner No late snacks Three meals on time Excessive supplements are not helpful Meal management/plan Having breakfast Maintaining regular weight Balanced diet	Adhering to a healthy diet	Healthy dietary habits
Korean food is healthy in general Not eating out Mistrust of cleanness of restaurant foods Homemade Korean food is healthy Prefer Korean foods	Insist on Korean food	
Difficult to obtain fresh ingredients Easy to consume meats	Access to certain ingredients	
Physical symptoms appear with small stress Hard to relieve stress	Vulnerable body to stress	Vulnerable physical and mental health
Notable weakness after a serious illness Indigestion Prolonged sedentary time Aged blood Poor blood circulation Narrowed sight angle Prolonged reactive time Reduced amount of meals	Weakness in body function	
Forgetful frequently Need to think longer than before	Deterioration in memory	
Hormonal change Lack of understanding Getting Stubborn	Changes in personality	
Hard to find pleasure Nothing is intriguing or fun	Decreased happiness	
Pets Grandchildren Baby from babysitting	Object of pleasure	Efforts to find vitality
Gratitude with work even in old ages Organized daily life by work	Pleasure of work	
Watching Korean TV shows Driving around Force work out Gospel Stroll Crying to overcome sorrow and depression	Efforts for better mood	
Remind the importance of healthcare Trying to execute healthy lifestyle	Conscious self-health care	
Can feel the air difference compared to Korea Nice air is the most favorable thing here	Nice air in the living area	Different environment compared to Korea
Easy to drive Lack of proper sidewalk Threatening of unleashed big dogs	Road environment	
Keep conflicting with spouse Personality change	Stress from spouse	Narrow personal relationship with Koreans
Limited gathering/chatting chances with Koreans Less social activity chances compared to Korea Less stress comes from interpersonal relationship	Lack of interactions among Koreans	

## Category 1: limited available health resources

The majority of OKAIs mentioned the limited accessibility to clinics or hospitals, lack of health resources for Korean seniors, and indiscriminating health information. They strongly pointed out that there needs to be at least one Korean doctor for Korean immigrants in a community where there are so many Korean immigrants. Setting up an appointment and the process of obtaining an appointment was also a barrier for OKAIs and caused them to give up visiting clinics since there is often a long waiting time to get an appointment and once at the clinic, a lot of paperwork in English is required before meeting the doctor. Other factors noted from the interview include the lack of health seminars for Korean immigrants and exercise centers or facilities with exercise equipment around the local area. Participants mentioned that family or friends are often their primary source of health information, or they rely on online searches using their cell phones rather than seeking advice from experts. As a result, the reliability and accuracy of the health-related information they acquire can fluctuate, occasionally leading to potential risks.

The only reason that I want to leave this community is that there are no Korean medical clinics.” (P7: Female, aged 66 years, married).

In Korea, medical insurance covers most of the examinations, and the access to clinics is very easy, however, here, the system makes a person more ill. (P4: Female, aged 66 years, married).

My uncle taught me this remedy called ‘blood clearing’. You create multiple small punctures in the painful area using several needles and suck out dead stagnant blood by cupping. It’s very helpful and easy to do at home. (P2: Male, aged 66 years, married).

## Category 2: limited English skill

OKAIs admitted that, due to their limited English proficiency, they expect lesser treatment or discrimination when meeting with clinicians. They often encounter significant frustration and stress when attempting to communicate with the clinic staff. As a result, they may choose to avoid visiting the clinic entirely and endure their symptoms rather than scheduling appointments. This language barrier contributes to misunderstandings and a lack of trust in both the staff and the doctor. OKAIs usually depend on interpretation from their English-proficient children or friends, but this is not always effective when it comes to unfamiliar medical terminology. OKAIs stress the need for interpreters during clinic visits and call for greater cultural understanding between the immigrant population and medical staff.

I just leave the clinic even if I have questions about doctors’ opinions or bills. (P2).

I really want to visit a clinic but am hesitant due to my English skill. I don’t want to bother someone to help me, so I just take the medicine by myself and bear the pain. (P7).

The doctor told me that I need MRI to help diagnose pain that is relatively mild. I couldn’t help but wonder if they would have made the same diagnosis if I were an American. (P4).

## Category 3: healthy dietary habits

OKAIs were generally found to have a relatively good knowledge of dietary habits, and they hold the strong belief that Korean cuisine is healthy. They mentioned all the habits they (try to) follow, such as having dinner early, the importance of having breakfast, not having late snacks, eating balanced meals on time, and maintaining a regular weight. However, some individuals expressed skepticism regarding the effectiveness of artificial supplements and concerns about the idea of consuming numerous daily supplements. There was a strong preference for food choices; they regarded Korean food as healthy and preferred homemade meals over American food or dining out. Nevertheless, they noted the challenge of sourcing fresh ingredients commonly used in Korean cuisine, such as various types of fish and vegetables.

I think America is good for people who like meats. Other than that, most of the ingredients are frozen and there aren’t many ingredients to choose from here to make Korean cuisine since there’s no big Korean grocery store. (P1: Female, 76 years, widowed).

My mother-in-law passed away at the age of 93 and she never took any medicine or supplements, nor did she ever go to the hospital. However, she always said, ‘Meal is the medicine’ and made sure to eat three well-timed meals each day. Why bother to take all the medications? (P7).

We seldom eat out as I believe that a healthy diet is just consuming fewer instant foods and minimizing eating out. Instead, I make Korean food at home with clean and fresh ingredients, without using too many artificial spices. Korean food is the best and healthy. (P6: Female, aged 65 years, married).

## Category 4: vulnerable physical and mental health

As with all older adults, OKAIs experience physical and mental health changes associated with aging. These changes include deteriorating memory, prolonged sensory processing, weakened body functions such as slower digestion, and long periods of sedentary behavior. Even minor stress can trigger a more pronounced physical response that takes longer to recover from than in previous years. Additionally, when dealing with serious illness, OKAIs experience a lower level of resilience, increasing the likelihood of becoming frail. Changes in mood and personality, as well as decreased happiness, can also negatively impact their mental health.

I thought I was very generous for my whole life but seem to become narrow-minded as I get older. I get stuck to minor words or things and get angry. (P5: Female, aged 72 years, married).

Frailty comes closer when going through seriously ill. I felt my resilience and energy decrease considerably. (P8: Female, aged 65 years, widowed).

## Category 5: efforts to find vitality

Despite having limited resources compared to living in Korea, OKAIs strive to maintain their vitality by utilizing the available resources around them. For instance, pets, grandchildren, and children they babysit can provide a sense of vitality in their daily lives. Additionally, working can help them maintain a regular lifestyle and provide a sense of purpose. They also engage in activities such as watching Korean TV shows, taking walks, driving, listening to worship music, and exercising regularly to improve their mood. One participant mentioned crying as an effective way to express their emotions and relieve stress when necessary. Moreover, OKAIs recognize the importance of health management and strive to maintain a healthy lifestyle.

One good thing about living in the U.S. is that I can still work even though I am old. It gives me energy and a daily routine, and I think this helps to maintain my health. (P2).

I soothe my homesickness by watching Korean TV shows. (P9: Female, aged 77 years, married).

I rely on my cat a lot, maybe more than my daughter. It seems that she understands my feelings. My cat gives me much relief and I even talk with my cat. (P8).

## Category 6: different environment compared to living in Korea

Compared to Korea, the clean air and less congested driving environment in the local area were commonly seen as positive factors of living in America. However, the lack of proper sidewalks was identified as a negative factor that hindered walking, which is detrimental to health. Another factor that limits walking is encountering large, unleashed dogs. OKAIs feel threatened and afraid when they come across unleashed dogs while walking in parks.

If I have to leave the States, I will miss the air and sky here. I could feel the difference in the air quality as soon as I inhaled air in Korea after getting off the plane at the airport. I start coughing soon after.” (P3: Female, aged 65 years, married).

Moreover, female participants strongly agreed that living in the U.S. had a positive impact on their stress levels as they were able to live apart from their family and relatives and did not feel obligated to take care of events for both their in-laws and their own families.

Being a woman in Korea is quite stressful because we are expected to take care of everything in both families. Additionally, being able to go out in comfortable clothes without dressing up is another aspect of reducing stress. I think these are privileges of living in America. (P4).

## Category 7: narrow personal relationship with koreans

Due to a lack of opportunities to interact with other older Koreans, they expressed the need for organized social activities or gatherings for Korean seniors at the community level for their healthy social life, which can positively impact their mental health. Nevertheless, due to restrictions on gatherings, particularly in the aftermath of COVID-19, OKAIs have mentioned that they are now spending more time with their spouses. Female participants, in particular, noted an increase in stress from their spouses as they age and have emphasized the significance of interpersonal relationships and their impacts.

A close person like a spouse can cause the most stress. When we were young, we did not fight a lot but after my husband’s retirement and spending more time at home together, problems arose. Maybe it’s due to changes in hormones? (P6).

The interrelationship between individuals appears to have a significant impact on mental health as getting older. While there is a need for more gatherings for Korean seniors for vitality, it is also worth noting that the lack of such gatherings can sometimes be less stressful. (P7).

## Discussion

This study examines the factors that contribute to frailty among older Korean American immigrants (OKAIs) and identifies limited English proficiency (LEP) as a significant obstacle to accessing healthcare services, potentially resulting in frailty. OKAIs face difficulties in making appointments, communicating with clinicians, and understanding explanations from their providers due to their LEP, all of which limit their health care seeking behavior. Except for big cities like Los Angeles, New York, and Atlanta, where Korean communities are well-organized, there are few Korean clinics available for LEP Koreans to visit. This language barrier means that OKAIs rely on information sources other than providers, such as friends or the internet. Potentially, it can lead to missed opportunities for treatment, delay treatment, or contribute to mistreatment. Although some participants have support from their children or friends, they still insist on the need for a translator with medical knowledge when seeking medical care.

The survey results of this study also support the negative effect of LEP on health literacy. There was a significant gap between the participants’ health literacy levels in English and Korean. While they possess good health literacy in their native language, their health literacy levels are significantly low when measured in English. This indicates that limited English proficiency hinders their health literacy levels in the US. However, all participants were aware of the importance of managing their health and had a good knowledge of healthy food or habits. Although they made efforts to live their daily lives in a healthy way, they were passive about visiting the clinic or hospital to meet their medical needs.

This study also found that most participants were getting regular health checkups, either by driving to a nearby big city where Korean clinics are located, flying to Korea to get their check-ups, or visiting clinics with the help of their children or friends who speak English. However, language barriers when seeking healthcare remain a significant issue for OKAIs who do not live in large cities. To enhance the clinic visit experience and alleviate the adverse impact of low health literacy in English, it is imperative to seek solutions for overcoming language barriers. This may involve the implementation of medical translators or the utilization of technology for translation and communication. These measures are essential as many OKAIs may have limited capacity for learning a new language in their later years. In this study, seven out of nine participants answered that they have trouble handling paperwork and speaking simple sentences despite living in the States for around 19 years. This result is similar to Jang et al.‘s (2016) study, which showed that 71% of OKAIs aged 60 or older have LEP [41]. Studies have identified translation systems, translation aids, visual aids, and plain language to enhance health literacy for LEP populations [42]. Some hospitals have also provided language services for those with LEP to improve communication and the quality of care [42]. Considering that long periods of residency for OKAIs do not necessarily lead to improved English proficiency, it’s more beneficial to concentrate on community or institutional-level health interventions rather than emphasizing individual efforts to enhance their English proficiency. Moreover, to address the challenge of limited health resources in Korean, there is a need for reliable health information or sources available in the Korean language. These interventions could be conducted through health seminars or educational sessions designed to help OKAIs better comprehend the medical culture in the United States. These sessions would cover a range of topics, including potential health scenarios, the processes within the healthcare system, medical terminology, English-language documents they might encounter during clinic visits, information about insurance coverage, billing procedures, and the available health resources for seniors in the community. In this way, OKAIs will be able to enhance their health literacy and discern what information is reliable and trustworthy. This will not only result in enhanced individual health outcomes but also provide tailored health resources within immigrant communities, which will cater to the health needs and interests of OKAIs.

OKAIs show good knowledge of dietary intake and insist on eating Korean food. However, about half of the participants were at risk of malnutrition. A previous study that used the National Health and Nutrition Examination Survey (NHANES) indicated that OKAIs who have lived in the U.S. for an average of 16 years and an average age of 70, mainly consume Korean food. Their diets consist of lower total calorie, folate, calcium, and fiber intake, but exhibit higher sodium consumption compared to the recommended dietary reference intakes [43]. The results show that even with their long period of residency in the U.S., unlike young Korean Americans, their food preference and perceptions of Korean food have not changed much. Potentially, this is because they spent their adulthood in Korea, which predisposed their appetite for Korean food. Additionally, they mentioned the difficulty of finding fresh ingredients in their current location as compared to Korea and are limited in making Korean foods with existing ingredients. To prevent frailty, when providing dietary education, understanding OKAIs’ food preferences and cultural understanding is needed. One approach could be educating about possible substitute ingredients and the importance of reducing sodium intake [43] and increasing the consumption of nutrients that are lacking in Korean food.

OKAIs also discussed the general physical and mental changes that most older adults experience as they age. To maintain their health and vitality, they intentionally stay active by walking, driving, working, and so on. In addition, OKAIS generally agreed that the environmental differences between Korea and the US, especially better air quality in the US, contributed to an active lifestyle. However, aspects of the local environment were viewed as hindering outdoor exercise, such as big dogs off leash in the park and limited sidewalks – both contributing to increased car use. However, despite the limited walking spaces, older adults favor the local roads due to less traffic and improved driving conditions in comparison to Korea or other major cities. When considering these factors mentioned above, a lack of physical activity can be expected in this population. Three participants had 0 METs/week; however, the average was 600 METs/week, which is the recommended METs/week for older adults [44] meaning that the rest of the participants performed physical activities more than the recommended METs/week.

Lastly, OKAIs mentioned that even after COVID-19, all the social meetings and gatherings that they used to have been canceled, which makes them less connected and bored. Participants expressed a desire for more casual meetings and gatherings, as well as health seminars for OKAIs. The only secure relationship they had was with their spouse, yet they expressed that conflicts with their spouse resulted in stress. OKAI’s main social support comes from their adult children, even if they live with their spouse [45]. However, as their children have grown up and left the home, only their spouse remains. Therefore, programs specifically designed for spouses of seniors could be a potential solution to address social isolation and provide additional support for OKAIs. Additionally, OKAIs experience higher acculturative stress and lower social support, which can result in depressive symptoms [45]. While participants in this study showed no sign of depression, it is necessary to provide various social gathering opportunities for OKAIs and reinforce their existing social networks to prevent social isolation and depression.

The present study explored various influencing factors of frailty in OKAIs through a focus group interview. The participants in this study generally exhibited low acculturation and limited English proficiency and shared unique cultural characteristics and beliefs. While it is challenging to ascertain whether the factors identified in this study will have short-term effects on frailty, the findings can contribute to a better understanding of this population and establish a basis for future research. When designing future frailty programs for OKAIs, the factors identified in this study should be considered. However, it is important to consider the limitations of the study. Despite efforts to saturate the data, the study only included one group with nine participants from a specific suburban area, which may limit its representation of the broader OKAI population. Therefore, caution should be exercised when generalizing the findings to all OKAIs in the US. Additionally, most participants were female, as their husbands or male counterparts were predominantly at work. Therefore, conducting further investigations with multiple groups based on gender or level of English proficiency could provide more extensive data. Furthermore, collecting survey data from a larger sample size of OKAIs could yield statistically significant quantitative data.

## Data Availability

No datasets were generated or analysed during the current study.

## Abbreviations

OKAI: Older Korean American immigrant
